# Dietary para-aminobenzoic acid, uric acid, and antibiotics modulate the susceptibility of *Anopheles darlingi* and *Anopheles albimanus* to *Plasmodium berghei*

**DOI:** 10.3389/fcimb.2025.1712389

**Published:** 2025-12-02

**Authors:** Breno A. Costa, Raquel S. M. Godoy, Lucas Henrique-Gomes, Rosa A. G. Santana, Silvia C. B. Justiniano, Stefanie C. P. Lopes, Wuelton M. Monteiro, Gisely C. de Melo, Nagila F. C. Secundino, Paulo F. P. Pimenta

**Affiliations:** 1 Instituto René Rachou, Fundação Oswaldo Cruz, Belo Horizonte, Minas Gerais, Brazil; 2 Programa de Pós-Graduação em Ciências da Saúde, Instituto René Rachou, Belo Horizonte, Minas Gerais, Brazil; 3 Programa de Pós-Graduação em Medicina Tropical, Fundação de Medicina Tropical Heitor Vieira Dourado, Univesidade do Estado do Amazonas, Manaus, Brazil; 4 Unidade de Entomologia Nelson Ferreira Fé (UENFF), Fundação de Medicina Tropical Doutor Heitor Vieira Dourado (FMT-HVD), Manaus, Brazil; 5 Instituto Leônidas & Maria Deane, FIOCRUZ-Amazônia, Manaus, Brazil; 6 Fundação de Medicina Tropical Dr. Heitor Vieira Dourado, Manaus, Brazil

**Keywords:** infection susceptibility, oocysts, midgut barriers, American anophelines, mosquito immune system

## Abstract

**Background:**

Malaria, caused by *Plasmodium*, is transmitted to humans through the bite of infected *Anopheles* mosquitoes. *Anopheles darlingi* and *Anopheles albimanus* are primary vectors in South and Central America, with *An. darlingi* the main vector in the Amazon region, which has the highest malaria levels in the Americas. Notwithstanding advancements in comprehending *Anopheles -Plasmodium* interactions in malaria vectors, information regarding these processes in New World vectors is nonetheless scarce. The limited understanding of *An. darlingi* is mainly attributable to the absence of experimental models suitable for its study. Researchers frequently utilize murine *Plasmodium* species, such as *Plasmodium berghei*, owing to their convenience in laboratory settings beyond endemic areas. Specific chemicals consumed by mosquitoes can affect the efficiency of *Plasmodium* infection.

**Methods:**

This study evaluates the susceptibility of *An. darlingi* and *An. albimanus* to *P. berghei* under different post-infection treatments, including para-aminobenzoic acid (PABA), uric acid, and penicillin/streptomycin (Pen/Strep).

**Results:**

Dietary supplementation with PABA, whether administered alone or in combination, influences susceptibility in both species. *Plasmodium berghei* sporozoites were detected in the hemolymph but not in the salivary glands until 28 days post-infection.

**Conclusion:**

This study is the first investigation to demonstrate that post-infection dietary treatments might influence the susceptibility of American vectors to *P. berghei*, hence broadening the scope for research on infection dynamics and parasite management mechanisms.

## 1 Introduction

Infected female *Anopheles* mosquitoes (Culicidae: Anophelinae) spread malaria to humans by their bites. Approximately fifty percent of the global population is vulnerable, particularly in low-income nations. In 2023, over 505,000 cases were reported in the Americas, with Brazil (33%), Venezuela (26%), Colombia (21%), Guyana (6%), and Peru (4%) comprising roughly 90% of the total cases (World Health Organization, 2024). In 2024, Brazil documented more than 138,000 malaria cases, with over 99% occurring in the endemic Amazon region (Fundação de Vigilância em Saúde do Amazonas, 2024).

Thirteen predominant malaria vector species exist in the Americas, with *Anopheles albimanus* and *Anopheles darlingi* being the most critical vectors for human malaria transmission. *An. albimanus* serves as the primary malaria vector in Central America and the Caribbean, whereas *Anopheles darlingi* is extensively dispersed throughout South America and is the most proficient vector for malaria vivax in the Amazonian nations (Ministério da Saúde, 2019, 2020; World Health Organization, 2024). *An. darlingi* exhibits significant vulnerability to *Plasmodium vivax* infection under both natural and laboratory environments (Rios-Velásquez et al., 2013; Pimenta et al., 2015). Nonetheless, research on *P. vivax* encounters obstacles owing to the parasite’s specific affinity for reticulocytes, which impedes continuous cell cultures and restricts *in vivo* experiments using anopheline vectors (Bermúdez et al., 2018). This highlights the necessity for alternate models to examine the interactions between *An. darlingi* and *An. albimanus* with various *Plasmodium* species.

Murine malaria parasites, including *Plasmodium berghei* and *Plasmodium yoelii*, are frequently employed as alternative models to investigate vector–parasite interactions. These models allow researchers to discern *Anopheles*-specific responses to various malaria parasites and have uncovered critical immune mechanisms, including the functions of melanization and effector genes (Dong et al., 2006; Jaramillo-Gutierrez et al., 2009; Contreras-Garduño et al., 2015; Orfano et al., 2016; Simões et al., 2017). Furthermore, a recent study revealed that specific midgut epithelial cells in *An. stephensi* are essential for regulating *P. berghei* infection (Barletta et al., 2024). These findings highlight the significance of these models in comprehending the *Anopheles*–*Plasmodium* relationship.

Dietary supplementation with specific compounds can markedly affect *Plasmodium* development in mosquitoes. Para-aminobenzoic acid (PABA), a nutrient belonging to the B-vitamin family and integral to folate biosynthesis, is essential for the *in vitro* growth of *Plasmodium* and in several animal models, including mice and non-human primates (Hawking, 1954; Jacobs, 1964; Ferrone, 1977; Zhang et al., 1992; Kicska et al., 2003; Parra et al., 2021). Nonetheless, its impact on parasite development in mosquito vectors differs, as PABA was shown to enhance *P. yoelii* infection in *Anopheles stephensi* but had no effect on *Plasmodium falciparum* in either *Anopheles stephensi* or *Anopheles gambiae* (Peters and Ramkaran, 1980; Beier et al., 1994). Uric acid, an antioxidant, can impede parasite melanization, hence promoting *Plasmodium* infection in *An. gambiae* (Kumar et al., 2003). Antibiotics such as penicillin/streptomycin (Pen/Strep) contribute to the phenomenon, as the mosquito midgut microbiota can adversely affect parasite viability (Azambuja et al., 2005; Dong et al., 2009; Meister et al., 2009; Gendrin et al., 2015). These findings underscore the significance of certain chemicals throughout all phases of parasite growth. Notwithstanding their established biological action, the impact of these substances on *P. berghei* infection in *An. darlingi* has yet to be investigated. Also, the effects of particular combinations of these chemicals on the interaction between *An. albimanus* and *P. berghei* remain unclear.

This research assessed the influence of PABA, uric acid, and a Pen/Strep combination on the development of *P. berghei* in *An. darlingi* and *An. albimanus*, with the main objective to establish and validate a model to investigate the interaction between *An. darlingi* and *P. berghei*, confirming parasite viability and infectivity. *Anopheles albimanus* was included not merely as a control, but as a positive reference vector, given its well-known susceptibility to *P. berghei*. Importantly, the analyses performed with *An. albimanus* also provide valuable data to optimize future experiments involving this vector–parasite pair. This framework allows the study to focus on infection dynamics in *An. darlingi* while ensuring that observed outcomes reflect genuine parasite–vector interactions rather than experimental artifacts. We demonstrate for the first time that targeted post-infection dietary treatments can influence the susceptibility of these American vectors to *P. berghei* infection. These findings facilitate inquiries into vector-parasite interactions, especially regarding susceptibility, refractoriness, and mechanisms of parasite management.

## 2 Materials and methods

### 2.1 Ethical statement

The study procedure was approved by the Fiocruz Animal Care and Use Committee (CEUA-Fiocruz) under approval number LW-33/22.

### 2.2 Mosquito rearing

Mosquitoes of the species *An. albimanus* (Buenaventura strain) and *An. darlingi* (RAS strain isolated and colonized from São Gabriel da Cachoeira, Amazon) were reared at temperatures between 26 and 28°C and relative humidity levels of 70–80% under a 12-h light/dark cycle. Larvae were raised in unchlorinated water and fed daily with Tetramine Flakes and Tetra Marine granules fish food. Upon reaching the pupal stage, they were transferred to cages until adult emergence. Adults were provided with a 10% glucose solution on cotton balls *ad libitum* until they were 3–6 days post-emergence, at which point they were used in experiments.

### 2.3 Parasite maintenance and infection feeding

Cryopreserved *P. berghei* (GFP-ANKA strain)-infected mouse blood, obtained from a first passage of mice infected with sporozoites, was intraperitoneally administered to 5- to 6-week-old BALB/c mice. Parasitemia was measured by preparing blood smears, fixing them with methyl alcohol (IMPEX), staining them with a 10% Giemsa solution, and counting infected red blood cells under a light microscope. These infected mice were used to infect mosquitoes once the parasitemia reached 5–10% and there were 1.5–3 male gametocyte ex-flagellations per field, as assessed by light microscopy. Female mosquitoes (3–6 days old) were infected by direct feeding on anesthetized *Plasmodium*-infected mice. Each experimental group consisted of 40 mosquitoes per cage. Dietary treatments were initiated immediately after the infectious blood meal—once the mice were removed and unfed females were excluded—and maintained until the final day of analysis (28 days post-infection). In each experimental replicate, two infected mice were used as the blood source for all mosquito cages. Both mice exhibited similar levels of parasitemia and ex-flagellations, assessed immediately prior to the infectious feeding. All cages were exposed simultaneously to the same two mice for 10 minutes each, in random order, ensuring that mosquitoes from every cage fed on the same blood source. To increase the likelihood of feeding, mosquitoes were starved for 8 hours prior to the infectious meal. The experiment was repeated in three independent replicates, each using a distinct pair of infected mice. Fully engorged mosquitoes were maintained at 21°C and 70% relative humidity and were provided with glucose, penicillin (5,000 units)/streptomycin (5,000 μg/mL) (Pen/Strep; Gibco), uric acid (Sigma-Aldrich), and PABA (Sigma-Aldrich) according to the following treatment groups: (i) 10% glucose; (ii) 10% glucose + 1% uric acid; (iii) 10% glucose + 1% Pen/Strep; (iv) 10% glucose + 1% Pen/Strep + 1% uric acid; (v) 10% glucose + 0.5 g/L PABA; (vi) 10% glucose + 0.5 g/L PABA + 1% Pen/Strep; (vii) 10% glucose + 0.5 g/L PABA + 1% Pen/Strep + 1% uric acid. To minimize potential interference with mosquito physiology while promoting optimal parasite development, treatments were initiated only after the infectious blood meal. All treatment solutions were refreshed daily and maintained until the day of dissection.

### 2.4 Midgut dissection and oocyst quantification

Mosquito infection was assessed 7 days post-infection (dpi). Females were anesthetized at 4°C for one minute, and their midguts were dissected in PBS under a stereoscope (Zeiss Stemi DV4). Midguts were fixed in Zamboni solution (4% paraformaldehyde, 0.4% picric acid in PBS) for 30m, washed five times, and mounted in Mowiol (Sigma) on microscope slides for visualization and direct oocyst counting. Infection prevalence and intensity were determined by examining midguts under a fluorescence microscope (Zeiss Axio Imager.A2). Representative midguts were photographed using an iPhone 14 (Apple) digital camera.

### 2.5 Hemolymph and salivary gland analysis

Mosquito hemolymph and salivary glands were analyzed at 14, 18, 22, 26, and 28 dpi. These analyses were performed in independent experiments specifically designed to detect sporozoites, using cages that initially contained 80–90 mosquitoes per treatment. At 7 dpi, ten mosquitoes from each group were collected for midgut oocyst evaluation, and the remaining females were maintained for subsequent sporozoite detection. Hemolymph was extracted by injecting a solution into the mosquito thorax and allowing it to extravasate (Castillo et al., 2006). Slides were stained with 10% Giemsa solution for 20 minutes and visualized and photographed using an optical microscope (Zeiss Axio Imager A2). Hemolymph from ten mosquitoes per group was evaluated individually. For salivary gland analysis, mosquitoes were placed on a slide with medium (RPMI 1640), and the salivary glands were dissected under a stereomicroscope (Zeiss Stemi DV4). After extraction, the salivary glands were washed twice in the same medium to remove impurities. Pools of ten dissected salivary glands were transferred to 1.5 mL Eppendorf tubes containing 30 μL of RPMI 1640 medium and gently disrupted using a glass pestle. All homogenates were analyzed in a Neubauer chamber under a light microscope. Additionally, some salivary glands were directly examined under light microscopy (Zeiss Axio Imager A2).

### 2.6 Statistical analysis

Statistical analyses were performed using GraphPad Prism software (version 8). Oocyst medians were compared using the non-parametric Mann–Whitney test, and infection rates were analyzed using the chi-square test.

## 3 Results

### 3.1 Impact of daily supplementation of uric acid, penicillin-streptomycin, and PABA on the susceptibility of *An. albimanus* and *An. darlingi* to *P. berghei*

We assessed *P. berghei* viability using *An. albimanus*, a vector known to be susceptible to this parasite (Frischknecht et al., 2006; Simões et al., 2017; Claudio-Piedras et al., 2020; Maya-Maldonado et al., 2022) and commonly used as a positive control for infection studies. To standardize experimental conditions, all infections and treatments were conducted concurrently, with both mosquito species being fed on the same *P. berghei*-infected mice.

**Figure 1:**
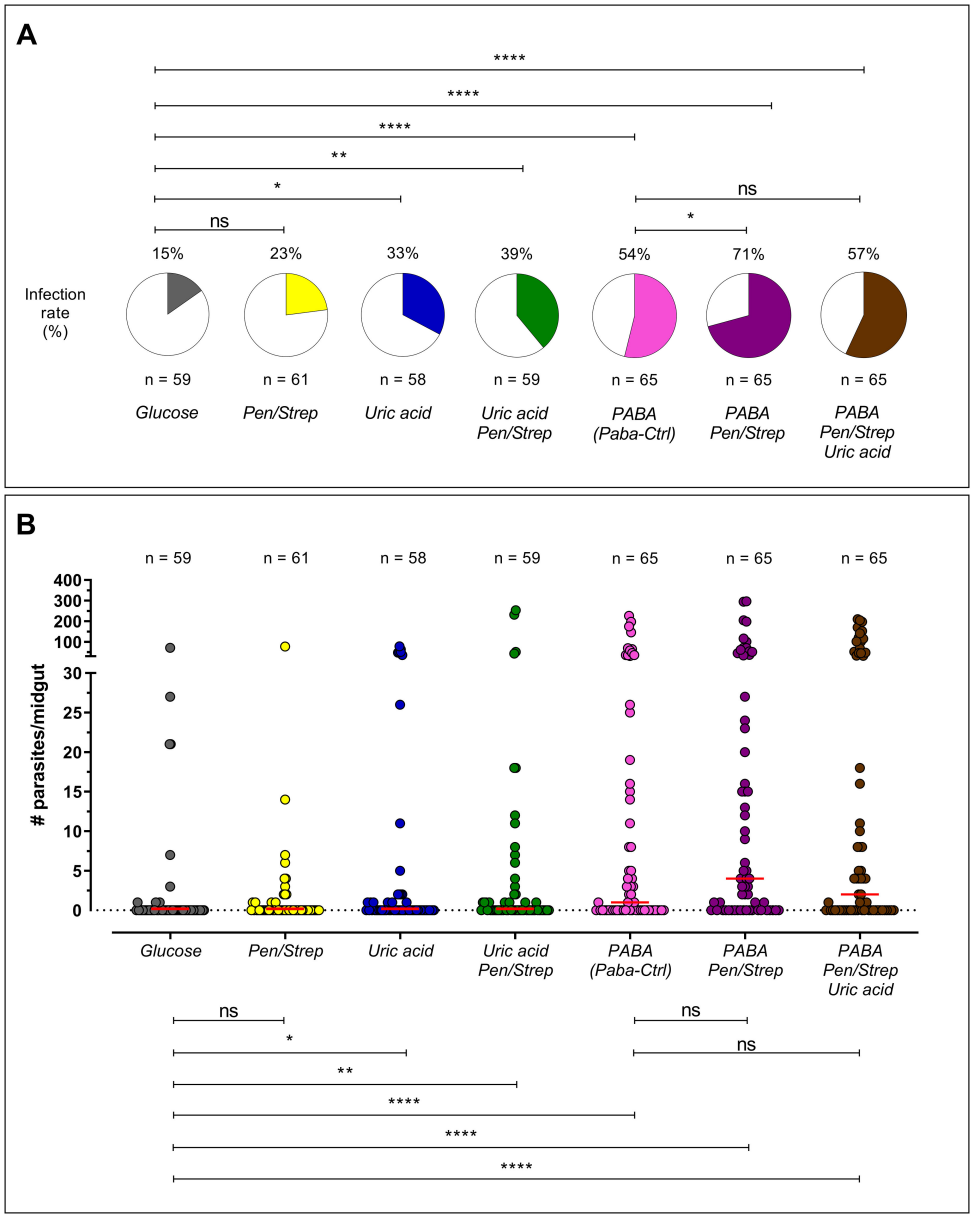
Infectivity of *An. albimanus* to *P. berghei* following the addition of various compounds as daily supplements to a sugary diet. The graph displays the infection rate **(A)** and the intensity of the infection **(B)**. Each point on the graph represents the number of oocysts found in an individual midgut 7 days after infection. The red line indicates the median number of oocysts. This graph shows the combined results of three independent experiments. ‘ns’ stands for no significance, and ‘Paba-Ctrl’ refers to the Paba Control. * (p < 0.05); ** (p < 0.005); **** (p < 0,0001).

**Figure 2:**
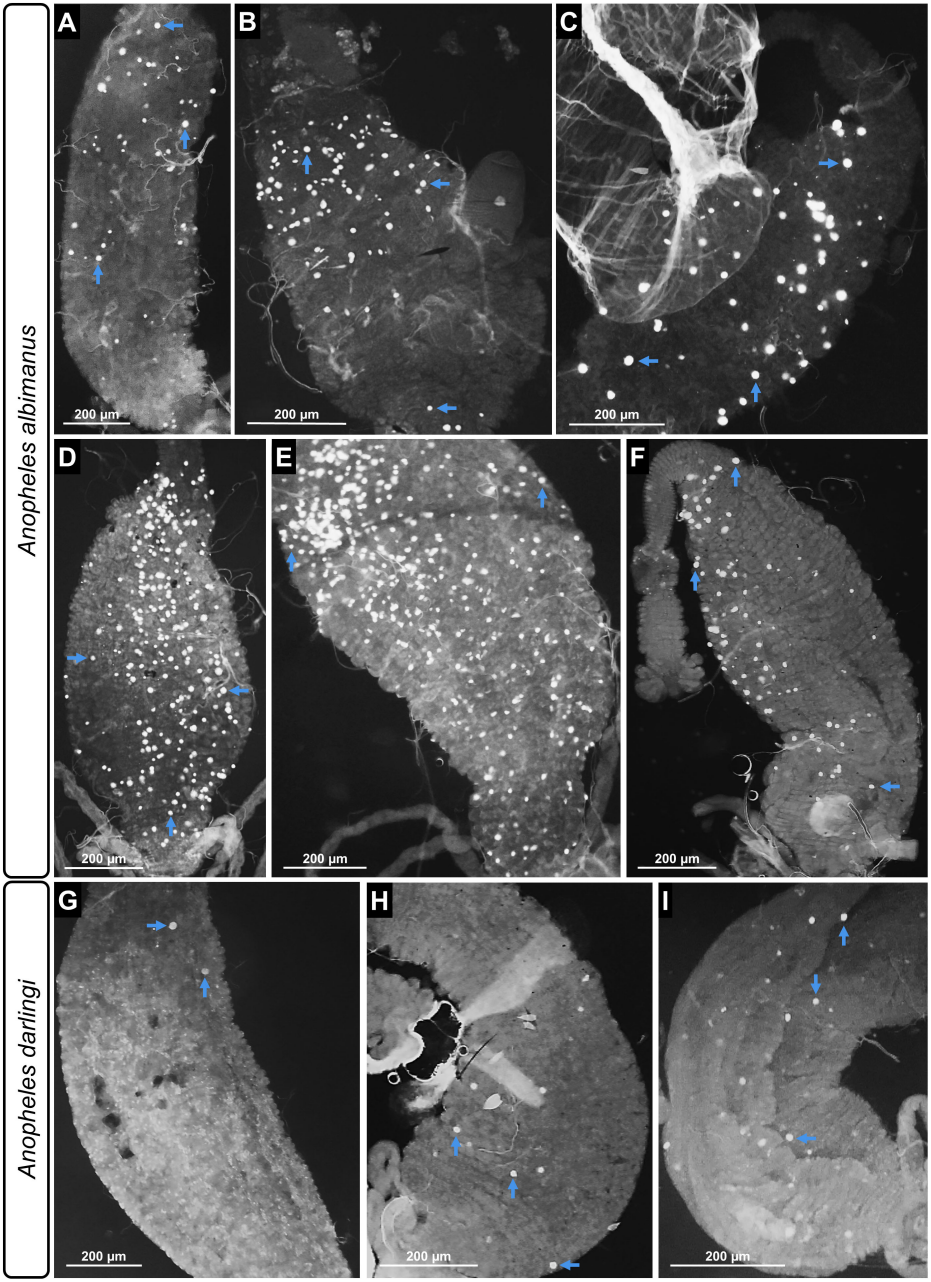
Effect of treatments on the intensity of *P. berghei* infection in *An. albimanus* and *An. darlingi*. The images display midgut infections after various treatments: control (glucose) **(A)**, uric acid **(B)**, uric acid + Pen/Strep **(C)**, PABA **(D)**, PABA + Pen/Strep **(E)**, and PABA + uric acid + Pen/Strep **(F)** in *An. albimanus*; and control (glucose) **(G)**, PABA **(H)**, and PABA + Pen/Strep **(I)** in *An. darlingi*. Blue arrows indicate specific oocysts. 10× magnification; scale bars: 200 µm.

In the analysis of infection rate, the groups treated with uric acid (33%; p = 0.01), uric acid + Pen/Strep (39%; p = 0.002), PABA (54%; p < 0.0001), PABA + Pen/Strep (71%; p < 0.0001), and PABA + Pen/Strep + uric acid (57%; p < 0.0001) showed a significantly higher number of infected mosquitoes compared to the control group fed with glucose. The most effective treatment was PABA + Pen/Strep, resulting in 71% of mosquitoes being infected, in contrast to only 15% in the control group (**Figure 1A**). We additionally analyzed infection intensity subsequent to post-infection treatments. Once again, the groups treated with uric acid (p = 0.04), uric acid + Pen/Strep (p = 0.005), PABA (p < 0.0001), PABA + Pen/Strep (p < 0.0001), and PABA + Pen/Strep + uric acid (p < 0.0001) had significantly higher numbers of parasites per midgut compared to the control group (**Figures 1B**, **2A–F**).

Upon examining the group administered PABA in conjunction with all compounds, and using mosquitoes treated solely with PABA as an independent control group (designated as Paba-Ctrl), we noted a significantly elevated infection rate when Pen/Strep was incorporated into Paba-Ctrl (71%; p = 0.04). Nevertheless, the incorporation of both Pen/Strep and uric acid into PABA-Ctrl did not yield any notable variations in infection intensity (**Figures 1A, B**).

**Figure 3:**
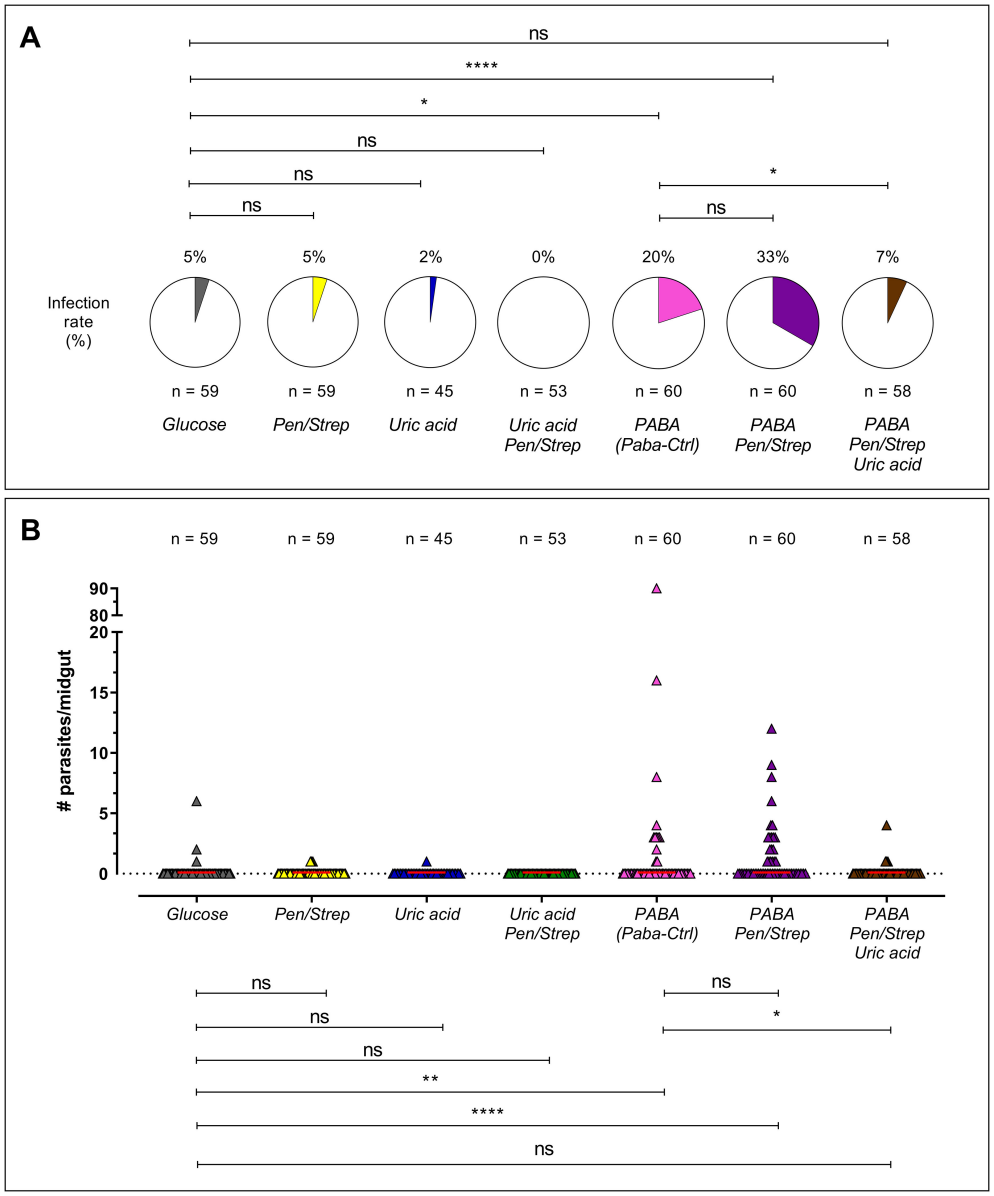
Analysis of various treatments in *An. darlingi* following infection by *P. berghei*. The figure illustrates the infection rate **(A)** and infection intensity **(B)**. Each data point on the graph represents the number of oocysts found in an individual midgut 7 days post-infection. The red line denotes the median number of oocysts. ns, no significance; Paba-Ctrl, Paba Control. This graph reflects the combined results from three separate experiments. * (p < 0.05); ** (p = 0,009); **** (p < 0,0001).

The analysis of identical parameters and treatments was subsequently broadened to *An. darlingi*. The control group, comprising mosquitoes fed with glucose, exhibited an infection rate of only 5%. Infection rates rose markedly in the groups administered PABA (20%; p = 0.009) and PABA + Pen/Strep (33%; p < 0.0001) (**Figure 3A**). In terms of infection intensity, the PABA (p = 0.01) and PABA + Pen/Strep (p < 0.0001) groups exhibited a considerably greater quantity of oocysts per midgut in comparison to the control group (**Figures 2G–I**, **3B**). The incorporation of Pen/Strep to the PABA treatment did not modify infection parameters compared to the group treated exclusively with PABA alone (Paba-Ctrl). The incorporation of uric acid into the PABA + Pen/Strep combination markedly diminished both the infection rate (7%; p = 0.03) and the infection intensity (p = 0.02) in comparison to Paba-Ctrl (**Figures 3A, B**). Data from all biological replicates of both *An. albimanus* and *An. darlingi* are provided in **Supplementary Table 1**.

**Figure 4:**
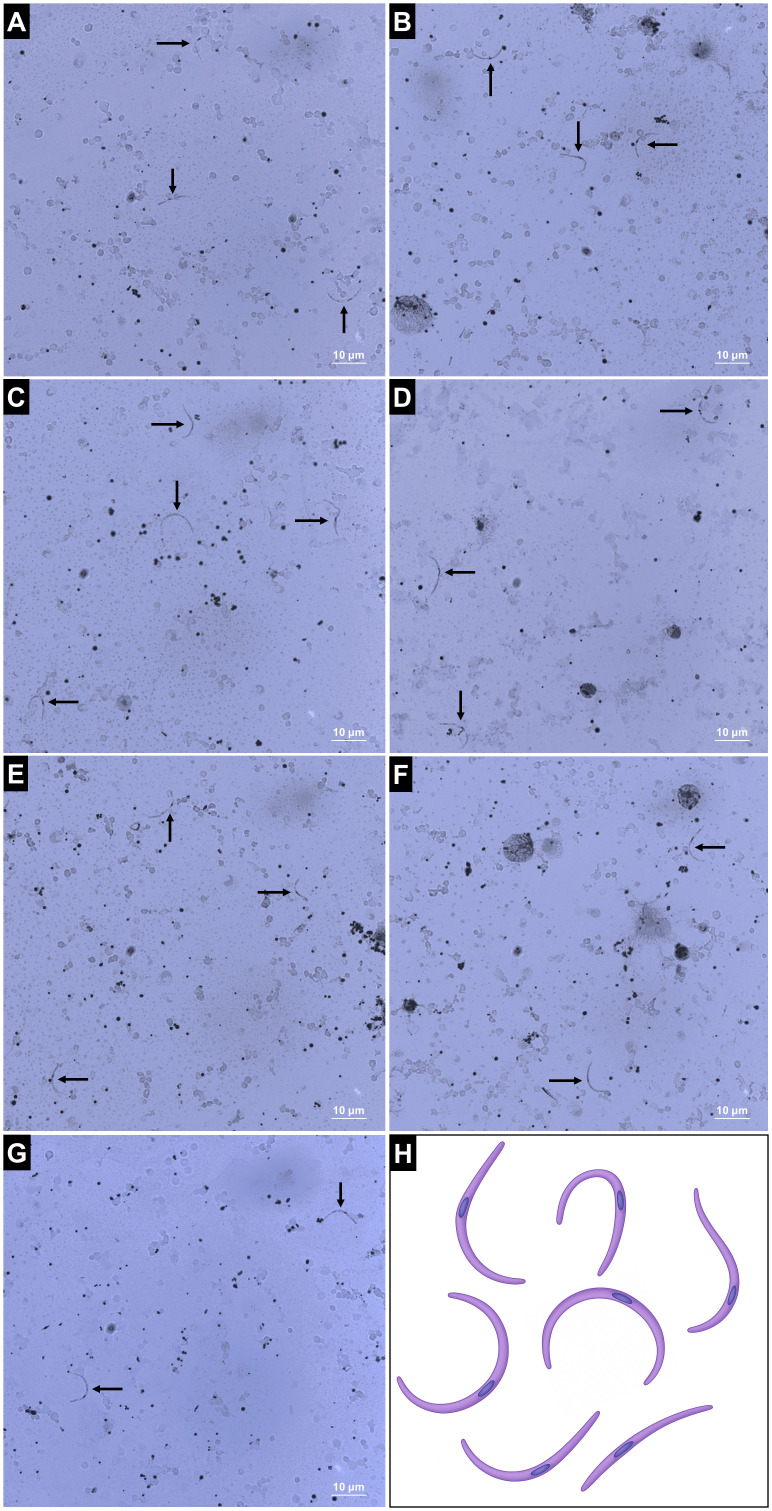
Dynamics of *P. berghei* sporozoite presence in the hemolymph of *An. albimanus* and *An. darlingi* following various treatments. Sporozoites were photographed in the hemolymph at 28 days post-infection (dpi) under the following treatment conditions: uric acid **(A)**, uric acid + penicillin/streptomycin (Pen/Strep) **(B)**, PABA **(C)**, PABA + pen/strep **(D)**, and PABA + uric acid + pen/strep **(E)** in *An. albimanus*; and PABA **(F)** and PABA + pen/strep **(G)** in *An. darlingi*. Illustration of sporozoite morphologies identified in the hemolymph of both mosquito species **(H)**. Created by the authors. The parasites demonstrate an elongated crescent shape, as indicated by the black arrows. Magnification: 63x; scale bars: 10 µm.

Upon verifying the appropriate development of *P. berghei* oocysts in the midgut of both *P. berghei* and *An. darlingi* post-treatment, we evaluated oocyst viability by identifying sporozoites in the mosquito hemolymph. The selected treatment groups for study exhibited elevated infection levels: uric acid, uric acid in conjunction with Pen/Strep, PABA, PABA combined with Pen/Strep, and the combination of PABA, Pen/Strep, and uric acid in *An. albimanus*, along with PABA and PABA combined with Pen/Strep in *An. darlingi*. Sporozoites were identified in the hemolymph of both vectors from the 14th day post-infection to the conclusion of the analysis on the 28th day post-infection. Hemolymph photodocumentation was conducted solely at 28 dpi (**Figure 4**). The experimental groups used for sporozoite analysis, as well as their respective *P. berghei* infection rates and oocyst counts in *An. albimanus* and *An. darlingi*, are summarized in **Supplementary Table 2**.

## 4 Discussion

In the absence of any treatment, our findings demonstrate that *An. darlingi* display partial resistance to *P. berghei*, as evidenced by the low infection levels observed. Conversely, *An. albimanus* exhibited increased susceptibility to the parasite, aligned with other findings (Frischknecht et al., 2006; Simões et al., 2017).

The incorporation of chemical compounds to the sugar diet of both vectors markedly modifies their vulnerability to *P. berghei* infection. In *An. albimanus*, the combination of Pen/Strep, uric acid, and PABA enhanced infection parameters. In *An. darlingi*, the use of PABA alone or in conjunction with Pen/Strep increased infection levels. These data indicate that these chemicals markedly affect the growth of *P. berghei* in both Neotropical vectors.

The antibiotic combination influences the survival of *Plasmodium* by the midgut bacteria of *Anopheles* through various mechanisms, including the activation of the mosquito’s immune response, the production of toxins or enzymes, and the establishment of a physical barrier that obstructs the interaction between the parasite and the midgut epithelium (Pumpuni et al., 1993; Azambuja et al., 2005; Dong et al., 2009; Meister et al., 2009). Certain antibiotics have been linked to enhanced development of *Plasmodium* in African and Asian vectors during infections caused by *P. falciparum, P. berghei*, and *Plasmodium vinckei petteri* (Dong et al., 2009; Sharma et al., 2013; Gendrin et al., 2015, 2016). Gendrin et al. (2015) observed that Pen/Strep exacerbates *P. berghei* and *P. falciparum* infections in *An. gambiae*. Conversely, other research indicates that Pen/Strep supplementation did not confer any advantages to *An. darlingi* during *P. vivax* infection (Moreno et al., 2014). In both mosquito species, treatment with Pen/Strep alone did not significantly affect the infection parameters of *P. berghei*, suggesting that the sole application of this antibiotic is inadequate to alter the sensitivity of these Neotropical vectors to the parasite. Together, these findings indicate that the application of Pen/Strep to enhance *Plasmodium* development in mosquito vectors is not uniformly applicable, as vector-parasite interactions exhibit variability in their responses to such interventions. It is crucial to highlight that mosquitoes exhibit significant variability in their microbiota composition, influenced by various factors, including geographic origin and breeding places (Scolari et al., 2019; Steven et al., 2021; Mosquera et al., 2023). This heterogeneity substantially affects the composition of bacterial communities in the anopheline midgut, which may subsequently alter the growth of *Plasmodium* parasites in various vectors (Villegas and Pimenta, 2014; Pimenta et al., 2015). Considering these interactions, additional research on *An. albimanus* and *An. darlingi i*s necessary to assess the effects of Pen/Strep and other antibiotics on gut bacterial communities that may influence susceptibility to various *Plasmodium* species.

The uric acid exacerbated *P. berghei* infection in *An. albimanus* but did not significantly affect *An. darlingi*, indicating distinct physiological responses to uric acid between the two species, potentially linked to variations in oxidative balance or the modulation of *Plasmodium*-specific immune mechanisms. Variations in systemic reactive oxygen species (ROS) levels in mosquitoes affect the melanotic encapsulation response to *Plasmodium* (Kumar et al., 2003; Molina-Cruz et al., 2008). The impact of the antioxidant uric acid in *An. gambiae* seems to differ among strains, probably owing to physiological variations such baseline reactive oxygen species (ROS) levels. For example, while uric acid significantly inhibits melanization without influencing phenol oxidase (PO) activity in the *An. gambiae* refractory (R) strain (Kumar et al., 2003), this potent antioxidant does not affect *Plasmodium* infection in the unselected *An. gambiae* (G3) strain (Molina-Cruz et al., 2008). The two strains exhibit a large disparity in their synthesis of reactive oxygen species (Kumar et al., 2003; Molina-Cruz et al., 2008). Additional research on the functions of reactive oxygen species and parasite melanization in *An. albimanus* and *An. darlingi* is necessary to clarify this matter, since these immunological factors have demonstrated critical roles in the anti-*Plasmodium* response in other *Anopheles–Plasmodium* systems.

*An. aquasalis* is effectively infected with *P. yoelii* only following treatment with a combination of uric acid and Pen/Strep, while this therapy had no impact on *P. berghei* infections (Orfano et al., 2016). The combination of Pen/Strep and uric acid elevated all infection parameters in *An. albimanus* but did not affect *An. darlingi*. The results, in conjunction with the findings of Orfano et al. (2016), suggest that this treatment provokes a species-specific response in the three Neotropical vectors of the *Nyssorhynchus* subgenus, with its efficacy in augmenting infectivity contingent upon the particular vector–parasite pairing. Simultaneously, despite the phylogenetic relationship of these mosquitoes and their contact with the same parasite, their interactions are not consistent, as they might be influenced by their associated bacterial communities. For example, mosquitoes of the same species from varying settings may display divergent microbiota compositions (Scolari et al., 2019; Steven et al., 2021; Mosquera et al., 2023), as previously mentioned.

The significance of PABA in the development of *Plasmodium* within both vertebrate hosts and mosquito vectors has been extensively documented (Hawking, 1954; Jacobs, 1964; Ferrone, 1977; Peters and Ramkaran, 1980; Zhang et al., 1992; Beier et al., 1994; McConkey et al., 1994; Kicska et al., 2003; Parra et al., 2021). PABA supplementation markedly enhanced *P. berghei* infection in both *An. albimanus* and *An. darlingi*, underscoring its essential involvement in the establishment of this murine parasite within these American vectors. This compound is routinely utilized in additional research targeting elevated *P. berghei* infection rates in *An. albimanus* (Contreras-Garduño et al., 2015; Claudio-Piedras et al., 2020, 2021; Maya-Maldonado et al., 2021, 2022). Furthermore, the efficacy of PABA was previously established in the *An. stephensi–P. yoelii*, where heightened parasite infection was noted irrespective of whether supplementation commenced prior to or following infection (Peters and Ramkaran, 1980). PABA exhibited no impact on *An. stephensi* or *An. gambiae* during *P. falciparum* infections, regardless of the dosage supplied or the timing in relation to the infection (Beier et al., 1994). Subsequent research on the traits of anopheline infections caused by several *Plasmodium* species may elucidate the distinct physiological and metabolic necessities of each parasite.

In both *An. albimanus* and *An. darlingi*, the combination of PABA and Pen/Strep demonstrated the highest efficacy in augmenting *P. berghei* infection. Frischknecht et al. (2006) also shown that this combination enhanced *P. berghei* infection in a different *An. albimanus* population (STECLA strain). The concurrent application of uric acid, Pen/Strep, and PABA to enhance parasite load in *Anopheles* vectors has not been previously recorded. We demonstrate for the first time that the concurrent administration of these drugs can directly augment *P. berghei* infection in *An. albimanus*. The mixture of all three drugs reestablished *P. berghei* infection levels in *An. darlingi* to those seen in the untreated group. The underlying mechanism in this situation remains ambiguous; nevertheless, we suggest that interactions among the chemicals consumed during *An. darlingi* infection may have yielded an impact contrary to that anticipated and observed in *An. albimanus*.

The success of the *Plasmodium* life cycle relies on the migration of sporozoites to, and their invasion of the mosquito salivary glands. Upon maturation of oocysts in the midgut of *Anopheles* mosquitoes, thousands of sporozoites are released into the vector’s hemolymph, subsequently migrating to the salivary glands to perpetuate the parasite’s life cycle (Pimenta et al., 2015). Giemsa staining demonstrated distributed sporozoites in the hemolymph of *An. albimanus* and *An. darlingi*; however, parasites were absent in the salivary glands, indicating a possible impediment in sporozoite invasion.

Our findings underscore the intricacy of mosquito responses to parasite infections, as mosquitoes from varied populations may demonstrate divergent infection outcomes. Our study failed not identify *P. berghei* sporozoites in the salivary glands of *An. albimanus*, in contrast to the findings of Frischknecht et al. (2006), who reported the presence of *P. berghei*, albeit in minimal numbers, in the salivary glands of a distinct *An. albimanus* population (STECLA strain). The interactions between *Anopheles* and *Plasmodium* are considered specific to both species and parasites (Molina-Cruz et al., 2012; Simões et al., 2017). Moreover, the infection rates of natural *P. vivax* and *P. falciparum* sporozoites in *An. albimanus* are generally low, as evidenced by Hurtado et al. (1997) and Grieco et al. (2005). In the latter instance, no *P. falciparum* sporozoites were detected in the salivary glands of one *An. albimanus* population, whereas merely 2.2% of mosquitoes from another population were infected (Grieco et al., 2005). *An. albimanus* may have mechanisms that inhibit sporozoite invasion of the salivary glands, maybe involving its immune system’s activities. Prior research has demonstrated that *Plasmodium*-infected *Anopheles* mosquitoes produce many antimicrobial peptides and other immunological compounds within their salivary glands (Dimopoulos et al., 1998; Dixit et al., 2009). Simões et al. (2017) demonstrated that the leucine-rich repeat immune protein LRIM1, an element of the mosquito’s complement-like system known for its strong anti-*Plasmodium* efficacy (Osta et al., 2004; Povelones et al., 2009), is a crucial factor influencing the success of *P. berghei* sporozoite infection in the salivary glands of *An. albimanus* (Simões et al., 2017). The observed absence of *P. berghei* sporozoites in the salivary glands of *An. albimanus* in this study may be attributed to the mosquito’s robust immunological responses against the parasite. Additional research is necessary to ascertain the precise immunological elements accountable for the noted anti-*P. berghei* action.

Research indicates that 41% of *An. darlingi* obtained in Belize, Central America, contained *P. falciparum* sporozoites in its salivary glands (Grieco et al., 2005). The lack of *P. berghei* sporozoites in their salivary glands suggests that this vector may have mechanisms that inhibit *Plasmodium* invasion of this organ, potentially through immune responses or modifications in the adhesion and invasion proteins or receptors used by the parasite. Certain receptors, including Saglin, gSG1, and members of the SGS protein family, located in the salivary glands of *Anopheles*, are crucial for the invasion of this organ by parasites (Vlachou et al., 2006; Ghosh and Jacobs-Lorena, 2009; Ghosh et al., 2009). Consequently, two non-mutually exclusive scenarios can be posited: (i) *An. darlingi* may be devoid of specific receptors necessary for *P. berghei* to invade the salivary glands; or (ii) *An. darlingi* may have these receptors, albeit in a modified configuration that blocks *P. berghei* from effectively invading this organ. The hypotheses are corroborated by the absence of evidence in the literature regarding *P. berghei’*s invasion of the salivary glands of *An. darlingi*. Consequently, further research is necessary to elucidate the role of the mosquito immune system and potential changes in salivary gland constituents that could impede parasite invasion—an vital phase in the *Plasmodium* life cycle.

## 5 Conclusions

This study’s results indicate that various post-infective treatments of *An. albimanus* and *An. darlingi* influence their susceptibility to *P. berghei* infection. We demonstrate for the first time that the incorporation of PABA and the antibiotic Pen/Strep makes *An. darlingi* vulnerable to *P. berghei*. Furthermore, the presence of sporozoites in the hemolymph of this vector signifies that the oocysts maturing in the midgut are viable. Given the absence of sporozoites in the salivary glands, we propose the presence of a barrier in this organ that inhibits *P. berghei* invasion, necessitating further investigation for elucidation.

The experimental framework established in this study offers a robust and adaptable platform to address a wide range of questions concerning the *Plasmodium–Anopheles* interaction, with particular emphasis on *An. darlingi*, the principal malaria vector in the Amazon region. Given the inherent fragility and environmental sensitivity of *An. darlingi*, experimental studies involving this species remain technically challenging, which has contributed to the scarcity of research on its interactions with *P. vivax* and *P. falciparum* in Brazil. By successfully adapting this vector to infection with *P. berghei*, our model overcomes an important methodological gap and enables in-depth exploration of parasite invasion dynamics, vector immune responses, and midgut physiological processes. It provides a foundation for dissecting the molecular and cellular events underlying epithelial repair, immune modulation by gut microbiota, and antiparasitic responses such as nitric oxide and reactive oxygen species production. Furthermore, this system allows detailed investigation of oocyst development, host–parasite nutrient competition, and sporozoite egress regulation—key determinants of vector competence. Importantly, the natural refractoriness of *An. darlingi* to salivary gland invasion offers a unique opportunity to identify intrinsic transmission-blocking mechanisms. Thus, this experimental model broadens the scope of research that can be pursued on *Plasmodium–An. darlingi* interactions and establishes a valuable foundation for discovering novel targets to interrupt parasite development before it reaches the transmissible stage.

## Data Availability

The original contributions presented in the study are included in the article/**Supplementary Material**. Further inquiries can be directed to the corresponding author.
