# Supplementary material for: Dietary para-aminobenzoic acid, uric acid, and antibiotics modulate the susceptibility of Anopheles darlingi and Anopheles albimanus to Plasmodium berghei
Source: Front Cell Infect Microbiol. 2025 Dec 2;15:1712389. doi: 10.3389/fcimb.2025.1712389 (PMC12705643; doi:10.3389/fcimb.2025.1712389)
Supplement: Supplementary file 1 [file DataSheet1.pdf]

Supplementary Material

An. albimanus					
**Replicate**	**Compounds**	**Sample size**	**Mean (± SEM)**	**Median**	**P-values**
1	*Glucose*	n = 15	0	0	—
	Pen/Strep	n = 20	5.15 (±3.84)	0	0.021
	Uric acid	n = 10	0	0	> 0.9999
	Uric acid Pen/Strep	n = 15	3.93 (±1.17)	0	0.0169
	PABA (Paba-Ctrl)	n = 22	24.91 (±11.90)	1	0.0018
	PABA Pen/Strep	n = 20	25.50 (±10.71)	2.5	0.0006
	PABA Pen/Strep Uric acid	n = 20	30.05 (±74.11)	4.5	0.0003
	PABA (Paba-Ctrl)	n = 22	24.91 (±11.90)	1	—
	PABA Pen/Strep	n = 20	25.50 (±10.71)	2.5	0.6579
	PABA Pen/Strep Uric acid	n = 20	30.05 (±74.11)	4.5	0.542
**Replicate**	**Compounds**	**Sample size**	**Mean (± SEM)**	**Median**	**P-values**
2	*Glucose*	n = 19	0	0	—
	Pen/Strep	n = 16	0.5 (±0.37)	0	0.0856
	Uric acid	n = 20	2.1 (±1.80)	0	0.2308
	Uric acid Pen/Strep	n = 21	3.71 (±2.44)	0	0.0036
	PABA (Paba-Ctrl)	n = 22	11.68 (±4.28)	0	0.0007
	PABA Pen/Strep	n = 19	15.26 (±6.44)	2	< 0.0001
	PABA Pen/Strep Uric acid	n = 25	21.96 (±9.79)	0	0.0004
	PABA (Paba-Ctrl)	n = 22	11.68 (±4.28)	0	—
	PABA Pen/Strep	n = 19	15.26 (±6.44)	2	0.3455
	PABA Pen/Strep Uric acid	n = 25	21.96 (±9.79)	0	0.8141
**Replicate**	**Compounds**	**Sample size**	**Mean (± SEM)**	**Median**	**P-values**
3	*Glucose*	n = 25	6.12 (±3.10)	0	—

	Pen/Strep	n = 25	0.56 (±0.32)	0	0.0855
	Uric acid	n = 28	12.52 (±4.45)	1	0.1679
	Uric acid Pen/Strep	n = 23	23.52 (±14.54)	0	0.8379
	PABA (Paba-Ctrl)	n = 21	23.71 (±12.09)	3	0.0423
	PABA Pen/Strep	n = 26	45.62 (±16.80)	9	0.0006
	PABA Pen/Strep Uric acid	n = 20	40.45 (±15.37)	4	0.0204
	PABA (Paba-Ctrl)	n = 21	23.71 (±12.09)	3	—
	PABA Pen/Strep	n = 26	45.62 (±16.80)	9	0.0953
	PABA Pen/Strep Uric acid	n = 20	40.45 (±15.37)	4	0.6206
**All replicates**	**Compounds**	**Sample size**	**Mean (± SEM)**	**Median**	**P-values**
	Glucose	n = 59	2.59 (±1.35)	0	—
	Pen/Strep	n = 61	2.04 (±1.28)	0	0.3463
	Uric acid	n = 58	6.24 (±2.16)	0	0.0422
	Uric acid Pen/Strep	n = 59	11.49 (±5.81)	0	0.0053
	PABA (Paba-Ctrl)	n = 65	20.05 (±5.75)	1	< 0.0001
	PABA Pen/Strep	n = 65	30.55 (±7.78)	4	< 0.0001
	PABA Pen/Strep Uric acid	n = 65	30.14 (±6.99)	2	< 0.0001
	PABA (Paba-Ctrl)	n = 65	20.05 (±5.75)	1	—
	PABA Pen/Strep	n = 65	30.55 (±7.78)	4	0.079
	PABA Pen/Strep Uric acid	n = 65	30.14 (±6.99)	2	0.4811

An. darlingi					
**Replicate**	**Compounds**	**Sample size**	**Mean (± SEM)**	**Median**	**P-values**
1	*Glucose*	n = 22	0.27 (±0.27)	0	—
	Pen/Strep	n = 25	0	0	0.4681
	Uric acid	n = 17	0.05 (±0.04)	0	> 0.9999
	Uric acid Pen/Strep	n = 19	0	0	> 0.9999
	PABA (Paba-Ctrl)	n = 16	7.25 (±5.60)	0	0.0109
	PABA Pen/Strep	n = 16	1 (±0.42)	0	0.0284
	PABA Pen/Strep Uric acid	n = 12	0.33 (±0.33)	0	> 0.9999
	PABA (Paba-Ctrl)	n = 16	7.25 (±5.60)	0	—
	PABA Pen/Strep	n = 16	1 (±0.42)	0	0.7895
	PABA Pen/Strep Uric acid	n = 12	0.33 (±0.33)	0	0.1267
**Replicate**	**Compounds**	**Sample size**	**Mean (± SEM)**	**Median**	**P-values**
2	*Glucose*	n = 21	0.04 (±0.04)	0	—
	Pen/Strep	n = 24	0.08 (±0.05)	0	> 0.9999
	Uric acid	n = 10	0	0	> 0.9999
	Uric acid Pen/Strep	n = 15	0	0	> 0.9999
	PABA (Paba-Ctrl)	n = 23	0.60 (±0.38)	0	0.4031
	PABA Pen/Strep	n = 15	1.66 (±0.77)	0	0.0062
	PABA Pen/Strep Uric acid	n = 22	0.13 (±0.07)	0	0.6069
	PABA (Paba-Ctrl)	n = 23	0.60 (±0.38)	0	—
	PABA Pen/Strep	n = 15	1.66 (±0.77)	0	0.0942
	PABA Pen/Strep Uric acid	n = 22	0.13 (±0.07)	0	0.7991
**Replicate**	**Compounds**	**Sample size**	**Mean (± SEM)**	**Median**	**P-values**
3	*Glucose*	n = 16	0.12 (±0.12)	0	—
	Pen/Strep	n = 10	0.10 (±0.10)	0	> 0.9999
	Uric acid	n = 18	0	0	0.4706
	Uric acid Pen/Strep	n = 19	0	0	0.4571

	PABA (Paba-Ctrl)	n = 21	0.38 (±0.20)	0	0.2762
	PABA Pen/Strep	n = 29	1 (±0.44)	0	0.0844
	PABA Pen/Strep Uric acid	n = 24	0	0	0.4
	PABA (Paba-Ctrl)	n = 21	0.38 (±0.20)	0	—
	PABA Pen/Strep	n = 29	1 (±0.44)	0	0.3988
	PABA Pen/Strep Uric acid	n = 24	0	0	0.0402
**All replicates**	**Compounds**	**Sample size**	**Mean (± SEM)**	**Median**	**P-values**
	Glucose	n = 59	0.15 (±0.10)	0	—
	Pen/Strep	n = 59	0.05 (±0.02)	0	0.8076
	Uric acid	n = 45	0.02 (±0.02)	0	0.4725
	Uric acid Pen/Strep	n = 53	0	0	0.2454
	PABA (Paba-Ctrl)	n = 60	2.32 (±1.54)	0	0.0093
	PABA Pen/Strep	n = 60	1.16 (±0.30)	0	< 0.0001
	PABA Pen/Strep Uric acid	n = 58	0.12 (±0.07)	0	0.8066
	PABA (Paba-Ctrl)	n = 60	2.32 (±1.54)	0	—
	PABA Pen/Strep	n = 60	1.16 (±0.30)	0	0.1601
	PABA Pen/Strep Uric acid	n = 58	0.12 (±0.07)	0	0.0209

Supplementary Table 1. Effects of different dietary compounds on *P. berghei* infection in *An. albimanus* and *An. darlingi*. Sample size, mean (± SEM) and median oocyst counts, and corresponding *p*-values are shown for each treatment group. Glucose was used as the control, and PABA-Ctrl represents mosquitoes fed only with PABA, without antibiotic or uric acid supplementation. Data from each biological replicate are presented individually, as well as the combined results from three independent experiments.
