# Supplementary material for: Dietary para-aminobenzoic acid, uric acid, and antibiotics modulate the susceptibility of Anopheles darlingi and Anopheles albimanus to Plasmodium berghei
Source: Front Cell Infect Microbiol. 2025 Dec 2;15:1712389. doi: 10.3389/fcimb.2025.1712389 (PMC12705643; doi:10.3389/fcimb.2025.1712389)
Supplement: Supplementary file 2 [file DataSheet2.pdf]

Supplementary Material

An. albimanus				
**Compounds**	**Sample size**	**Midgut infection (infected/total)**	**Mean (± SEM)**	**Median**
Uric acid	n = 10	4/10	2.20 (± 1.06)	0
Uric acid<br>*Pen/Strep*	n = 10	4/10	12 (± 9.23)	0
PABA	n = 10	6/10	22.40 (± 10.59)	2
PABA<br>*Pen/Strep*	n = 10	5/10	9.20 (± 3.42)	5.5
PABA<br>*Pen/Strep*<br>*Uric acid*	n = 10	5/10	16.20 (± 6.61)	6

An. darlingi				
**Compounds**	**Sample size**	**Midgut infection (infected/total)**	**Mean (± SEM)**	**Median**
PABA	n = 10	3/10	1.10 (± 0.79)	0
PABA<br>*Pen/Strep*	n = 10	4/10	1.70 (± 0.90)	0

Supplementary Table 2. Dietary treatments and *P. berghei* infection in *An. albimanus* and *An. darlingi* used for sporozoite analysis. Mosquitoes were fed uric acid, PABA, or combinations with antibiotics (Pen/Strep) and were selected for the evaluation of sporozoite presence in the hemolymph and salivary glands. Sample size, midgut infection (infected/total), mean (± SEM), and median oocyst counts are shown for each treatment group. A total of 80–90 mosquitoes were infected, of which 10 were analyzed at 7 days post-infection (dpi) to assess midgut infection levels for each treatment. The remaining females were maintained and examined at 14, 18, 22, 26, and 28 dpi to detect sporozoites.
